# One dimensional edge localized YSR states in CrCl_3_ on NbSe_2_

**DOI:** 10.1038/s41535-025-00759-2

**Published:** 2025-05-27

**Authors:** Jan P. Cuperus, Arnold H. Kole, Andrés R. Botello-Méndez, Zeila Zanolli, Daniel Vanmaekelbergh, Ingmar Swart

**Affiliations:** Condensed Matter and Interfaces, Debye Institute for Nanomaterials Science, Utrecht University, Utrecht, The Netherlands

**Keywords:** Electronic properties and materials, Magnetic properties and materials, Superconducting properties and materials, Two-dimensional materials

## Abstract

Magnet/superconductor hybrid systems have been put forward as a platform for realizing topological superconductivity. We investigated the heterostructure of ferromagnetic monolayer CrCl_3_ and superconducting NbSe_2_. Using low-temperature scanning tunneling microscopy, we observe topologically trivial Yu-Shiba-Rusinov (YSR) states localized at the edge of CrCl_3_ islands. DFT simulations reveal that the Cr atoms at the edge have an enhanced *d*-orbital DOS close to *E*_*F*_. This leads to an exchange coupling between these atoms and the substrate that rationalizes the edge-localization of the YSR states.

## Introduction

The interplay between magnetism and superconductivity gives rise to a variety of exciting phenomena, of which the macroscopic Meissner effect is the most well-known. On the nanoscale, magnetic impurities in superconductors induce bound states known as Yu-Shiba-Rusinov (YSR) states^1–3^. YSR states do not only provide fundamental insight into the impurity problem in superconducting systems; they may also become the building blocks of quantum materials exhibiting topological superconductivity (TSC). Most notably, 1D TSC was induced in *s*-wave superconductors by chains of magnetic adatoms^4–7^. Along the same lines, 2D TSC can be induced in planar heterostructures of magnetic and superconducting materials^8–10^. Such heterostructures have been realized by depositing a monolayer (ML) of bulk magnets (Fe, Co) on *s*-wave superconductors (Pb, Re)^11,12^. Another approach is to employ magnetic van der Waals materials, such as Fe_3_GeTe_2_^13^, Cr_2_Ge_2_Te_3_^14^, or the chromium halides CrX_3_ (X = Cl, Br, I)^15–17^. In contrast to Fe and Co, these 2D magnets can be semiconducting (Cr_2_Ge_2_Te_3_) or insulating (CrX_3_). Thus far, heterostructures contained 2D magnets with an out-of-plane orientation, for which the emergence of an edge-localized state at zero bias is consistent with TSC^11,12,18,19^. In case of CrBr_3_/NbSe_2_ there is significant controversy: one research group found zero-energy boundary localized states^18^, attributed to Majorana modes, whereas another group found YSR-like states^20^.

Here, we report on a complementary heterostructure: CrCl_3_/NbSe_2_. ML CrCl_3_ is an in-plane ferromagnetic insulator, which is nearly lattice matched to NbSe_2_. Using scanning tunneling microscopy (STM) and spectroscopy (STS), we observe a YSR state localized at the edges of CrCl_3_ islands. We interpret our results via the exchange interaction between CrCl_3_ and NbSe_2_, as calculated using numerical simulations based on first-principles theory. The exchange energy is found to be stronger at the edge than in the CrCl_3_ interior. Our work shows that edge states in magnet/superconductor heterostructures can also be trivial in nature.

## Results

**Fig. 1:**
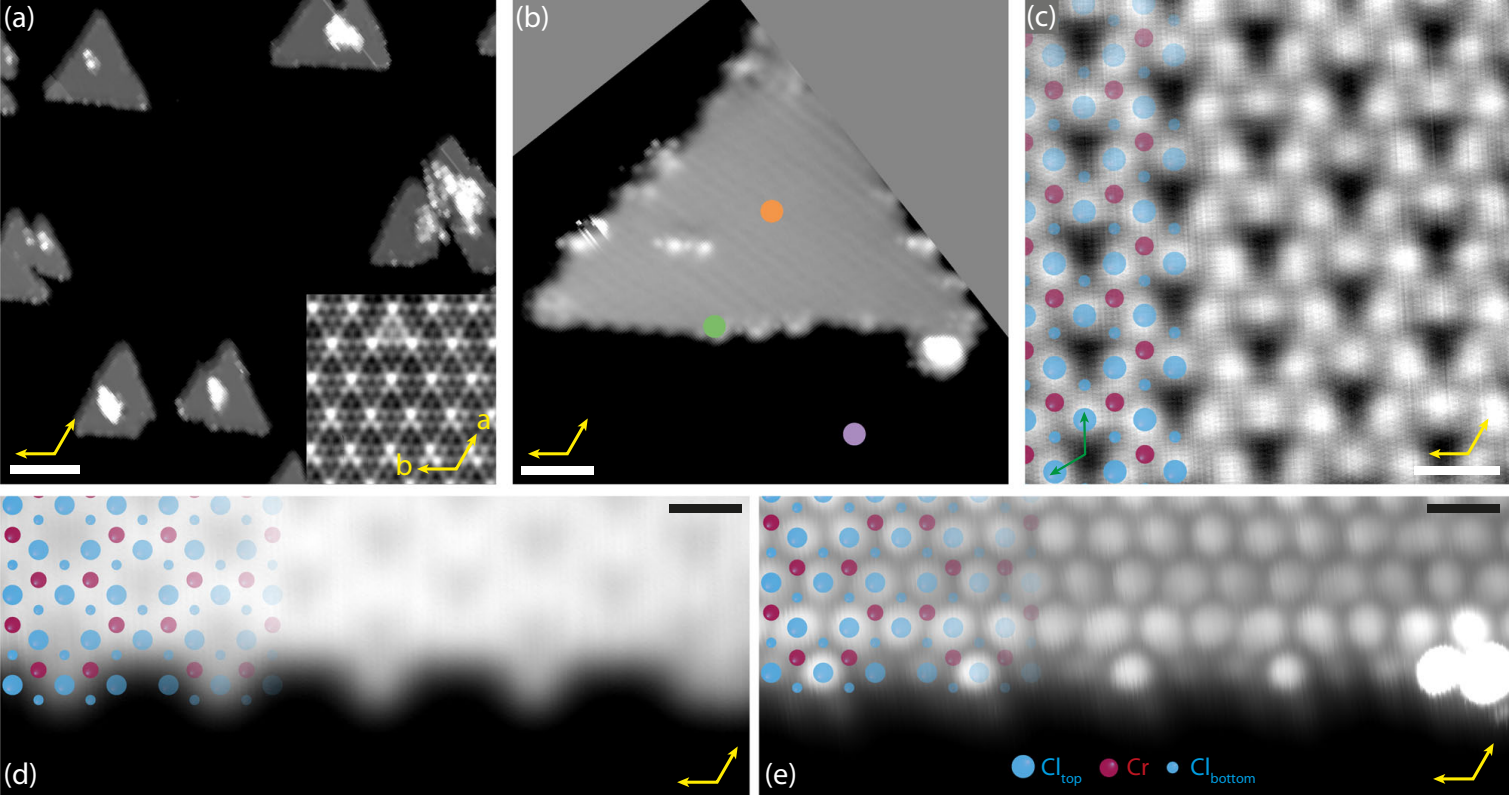
Morphology of as-grown CrCl_3_/NbSe_2_ heterostructure. **a** Large scale topograph of as-grown 1 ML CrCl_3_ islands on NbSe_2_ (0.8 V, 50 pA, scale bar: 20 nm). The inset shows the NbSe_2_ lattice, a and b denote the crystallographic directions of the 2H-NbSe_2_ (50 mV, 100 pA). **b** Topograph of a single island (0.8 V, 50 pA, scale bar: 3 nm). Pink, orange and green circles indicate dI/dV spectroscopy positions of Fig. 2a. **c** Small scale (constant height) image inside of a CrCl_3_ island, showing the lattice of Cl atoms in the top layer. Set point: 0.8 V, 50 pA; scale bar: 5 Å. A structural model of CrCl_3_ is overlaid. **d**, **e** Constant height image of an island edge, overlaid with a structural model. Set point (**d**): 0.8 V, 80 pA; set point (**e**): 5 mV, 500 pA; scale bars: 5 Å.

Figure 1a shows a topograph of as-grown CrCl_3_/NbSe_2_ heterostructures. CrCl_3_ has formed islands with a typical width of 10–30 nm, which all have the same orientation with respect to the substrate. The triangular shape of the islands indicates a clear preference for one type of edge termination (vide infra). The apparent height of the islands is 0.6 nm, similar to what has been reported for CrBr_3_/NbSe_2_^20^ and confirming that the islands consist of a single ML of CrCl_3_. In Fig. 1b, a zoom-in of a CrCl_3_ island is shown. Several imperfections are noticeable at the edges of the islands. A high-resolution STM image taken in the center of an island reveals the characteristic lattice formed by the top layer of Cl atoms (Fig. 1c). We measure a lattice parameter of 6.03 Å (±0.04 Å)^21^. The CrCl_3_ lattice is rotated by 30^∘^ with respect to the NbSe_2_ substrate. In this orientation, the lattice parameters of CrCl_3_ and NbSe_2_ match to within 0.4%, in agreement with the absence of moiré patterns in larger-scale images. Using STS experiments, the electronic properties of CrCl_3_/NbSe_2_ were investigated. This data, shown in Supplementary Fig. S1, shows that NbSe_2_ slightly dopes the CrCl_3_ and suggests that the charge-density wave of NbSe_2_ is retained beneath the CrCl_3_ islands.

From the atomically resolved image in Fig. 1c, combined with the relative orientation of the island, we conclude that the CrCl_3_ islands are terminated by the armchair direction of the Cr sublattice. This is confirmed by two constant-height STM scans of an island edge shown in Fig. 1d, e. In the scan at 0.8 V (Fig. 1d), the hollow sites in the Cr sublattice are observed and used to overlay the atomic model with the edge. By changing the applied bias, the atomic contrast could be changed. The image recorded at 5 mV (Fig. 1e) clearly shows the positions of the Cl atoms. In both images, the armchair termination of the Cr sublattice is evident. In the outermost line of Cl_top_ atoms, intensity variations can be observed: a Cl atom with two Cr neighbors shows a higher intensity than the Cl atoms with only one neighboring Cr. We speculate that these intensity variations are due to geometric relaxations that are inherent to edge formation.

**Fig. 2:**
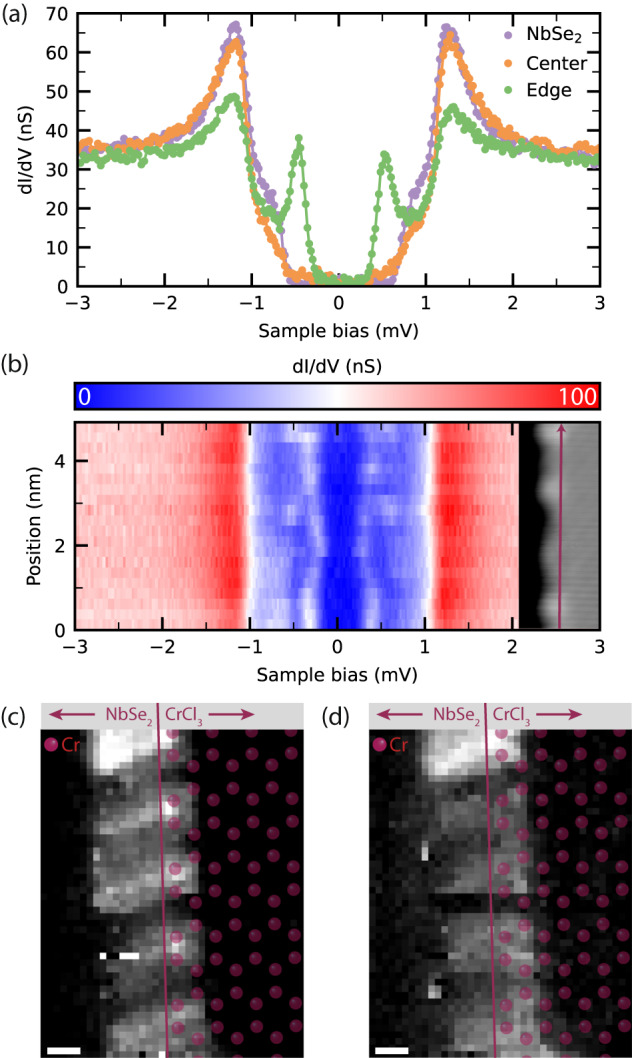
Spatially resolved electronic characterization of CrCl_3_/NbSe_2_. **a** Differential conductance spectra taken at the positions indicated in Fig. 1b. Set point: 5 mV, 150 pA. **b** Contour plot of dI/dV spectra taken along the edge segment highlighted in the inset. Set point: 5 mV, 250 pA; inset: 0.5 V, 80 pA; 6 nm × 15 nm. Spatial maps of the YSR states at +560 *μ*V (**c**) and -560 *μ*V (**d**), extracted from a grid spectroscopy experiment, conducted along the purple line shown in the inset of (**b**). As a guide to the eye, the atomic Cr sublattice has been overlaid with the data. Additionally, the edge termination is indicated by the near-vertical line. Set point: 5 mV, 300 pA; scale bars: 5 Å.

Next, we turn our attention to the electronic structure of CrCl_3_/NbSe_2_. The superconducting (SC) gap measured between the islands (Fig. 2a, purple curve) is the same as that of pristine NbSe_2_. STS data from the center of a CrCl_3_ island (orange curve) shows a SC gap size identical to NbSe_2_, with minor differences in the height of the coherence peaks and the shape of the gap. Interestingly, spectra acquired at the edge of CrCl_3_ islands show pronounced YSR states, see the green curve in Fig. 2a (peaks are symmetric in energy but asymmetric in amplitude). We observe these YSR states on all edges of each CrCl_3_ island (see Supplementary Fig. S2). We have scrutinized the YSR states using spatially resolved dI/dV spectroscopy. STS experiments along the edge of an island (Fig. 2b) reveal that the energy of the YSR state varies along the edge. The following two factors could be responsible for this observation: coupling between individual YSR states and changes in the local environment due to defects or disorder^22^. An example of the impact of the latter is the emergence of a second pair of YSR states, approximately halfway in the line spectrum in Fig. 2b. A different edge, with a different atomic structure, shows a different periodicity (see Supplementary Fig. S3). Note that the dependence of the in-gap state energy on the local atomic edge structure provides evidence of the trivial nature of the edge modes.

To elucidate the correlation between the YSR state amplitude and the CrCl_3_ lattice, we performed grid spectroscopy on a small edge segment. Figure 2c, d shows dI/dV-maps of the YSR state, in which a clear periodicity of ca. 1 nm can be observed. By means of a simultaneously acquired topography image, the atomic model of CrCl_3_ was overlaid with the dI/dV-map (see Supplementary Fig. S4). The intensity maxima are located between two outermost Cr atoms of the armchair edge. We note that these Cr atoms are coordinated to only two other Cr atoms, whereas bulk Cr atoms have three Cr neighbors. The spatial extent of the YSR states, perpendicular to the island edge, is exaggerated by a tip artifact.

**Fig. 3:**
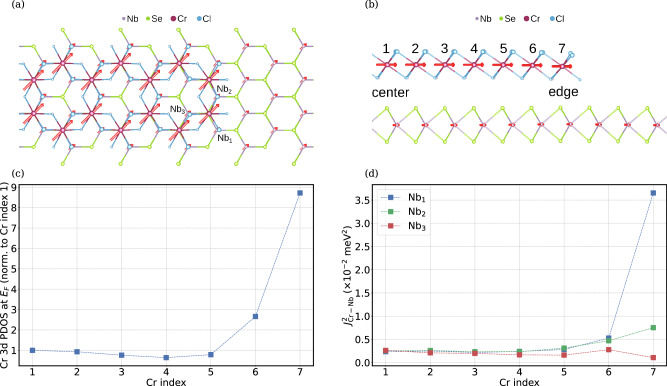
Theoretical calculations for CrCl_3_ nanoribbons on NbSe_2_ to model the edge of the triangular CrCl_3_ islands. Only results for the experimentally observed edge are shown. Geometry after relaxation shown from the top (**a**) and side (**b**). Mulliken magnetic moments of atoms are shown as red arrows. The numbering of the Cr atoms in (**b**) refers to the indices used in (**c**, **d**). The Cr atom at index 1 is in the center of the ribbon. **c** Integrated density of states in a region of width 4 meV centered on the Fermi energy, projected on the 3d orbitals of Cr (PDOS), for different Cr atoms when moving from the center of the ribbon to the edge. **d** Isotropic exchange squared *J*^2^ between nearest neighbor pairs of Cr and Nb atoms when moving from the center of the ribbon to the edge. Labels refer to the three different nearest Nb neighbors of each Cr as seen in (**a**).

To understand the origin of the experimentally observed in-gap states, we computed the electronic and magnetic properties of CrCl_3_/NbSe_2_ using density functional theory (DFT), as implemented in the SIESTA code^23,24^. The NbSe_2_ substrate was modeled using a single monolayer. To model the edge of the CrCl_3_ islands, we have used a nanoribbon with a width of 13 Cr atoms from edge to edge. In agreement with the experimental result, the ribbons were constructed with the armchair edge. The relaxed geometry is shown in Fig. 3, from the top (a) and from the side (b). Most notably, the Cr atoms at the edge bend slightly towards the substrate. In the center the Cr-Nb distance is 6.31 Å, while at the edge it is 6.04 Å. This suggests that the interaction between Cr and the substrate is stronger at the edge than in the center. In Fig. 3a, b, the Mulliken magnetic moments of each atom are shown as red arrows. All Cr atoms, including those at the edge, are ferromagnetically aligned in-plane and have a magnetic moment of *S*_Cr_ = 3.2 *μ*_B_ (i.e. the minor bending does not significantly affect the magnetic moment). The CrCl_3_ induces a magnetic moment in the Nb atoms of *S*_Nb_ = 1.0 *μ*_B_. The difference in energy between ferro- and anti-ferromagnetically coupled layers is within the computational accuracy (see Supplementary Discussion).

Next, we calculated the projected density of states (PDOS) of the 3d orbitals of the different Cr atoms in the ribbon (see Fig. 3c). The Cr atom at the edge has a notably higher PDOS close to *E*_*F*_ [PDOS(*E*_*F*_)], compared to atoms in the center of the ribbon. The PDOS(*E*_*F*_) drops off drastically when moving away from the edge. Already at the third Cr atom, the PDOS(*E*_*F*_) has reached the value it has in the interior. Presumably, the increased PDOS(*E*_*F*_) at the edge enhances the van der Waals attraction, causing the ribbon to bend. We speculate that the PDOS(*E*_*F*_) also plays a key role in the appearance of the edge-localized YSR states. To gain more insight into this, we computed the isotropic exchange *J* between nearest-neighbor pairs of Cr and Nb atoms, as a function of spatial position (Fig. 3d), using the TB2J code^25^. We find that the exchange interaction *J* is smaller in the center of the ribbon (~0.05 meV) than at the edge (~0.2 meV). The value of *J* is positive everywhere, indicating ferromagnetic coupling between the Cr and Nb atoms. Since the predicted edge bending is minor compared to the distance between the CrCl_3_ and the substrate, we expect the enhanced exchange coupling to be mostly due to the termination of the ribbon.

## Discussion

The differences in the exchange interaction can be used to rationalize the experimental observations in the classical spin model of YSR states^1–3,26^. In this model, the bound state energy *ϵ* depends on the exchange interaction *J* as:

1 $$\epsilon =\Delta \frac{1-{(JS\pi {\rho }_{s})}^{2}}{1+{(JS\pi {\rho }_{s})}^{2}}=\Delta \frac{1-{\gamma }^{2}{J}^{2}}{1+{\gamma }^{2}{J}^{2}},$$

where we have captured the impurity spin *S* and the substrate density of states at *E*_*F*_ in the normal state *ρ*_*s*_ in the parameter *γ* = *Sπρ*_*s*_. Using Δ = 1.25 meV and an energy *ϵ*_edge_ = 0.56 meV for the YSR state found at the edge, the exchange interaction *J* = 0.2 meV (*J*^2^ = 0.04 meV^2^) yields *γ* = 3.273 meV^−1^. With this value of *γ*, and the exchange interaction *J* = 0.05 meV (*J*^2^ = 0.003 meV^2^) in the center, we estimate *ϵ*_center_ to be 1.20 meV. This means that YSR states in the center of CrCl_3_ islands are positioned close to the coherence peaks, making it hard to detect them using STS with a metallic tip – in agreement with our experimental data (see Fig. 2a). For the above estimation, we have assumed the spin moment *S* of the Cr atoms and density of states *ρ*_*s*_ of NbSe_2_ to be identical for the center and edge of the CrCl_3_ island, which seems justified by our DFT simulations. Given the qualitative nature of the model, combined with the approximate character of the exchange-correlation potential used to calculate *J*, we focus only on relative changes in the magnitude of exchange coupling.

We note that different configurations of the CrCl_3_/NbSe_2_ heterostructure have very similar energies in our DFT simulations. In fact, the energy difference between ferro- and antiferromagnetically aligned CrCl_3_ and NbSe_2_ spins is smaller than the accuracy of DFT (~1–5 meV). Nevertheless, we find the same qualitative behavior irrespective of the exact configuration: at the edge, the interlayer distance decreases, PDOS(*E*_*F*_) increases, and the exchange interaction becomes stronger. For a more detailed discussion on the different structural and magnetic configurations, we refer to the Supplementary Discussion.

From the foregoing discussion, we conclude that the YSR edge state observed in CrCl_3_/NbSe_2_ originates from the partially unscreened magnetic moment of Cr. Hence, the edge state is topologically trivial. This is in stark contrast to CrBr_3_/NbSe_2_, where a zero-energy edge state is attributed to topological superconductivity^18^. Possibly, this contrast arises from the absence of a moiré pattern in CrCl_3_/NbSe_2_. It is thought that the topological superconductivity in CrBr_3_/NbSe_2_ is facilitated by the moiré pattern, which expands the suitable region of phase space^19^. Because no moiré pattern exists for CrCl_3_/NbSe_2_, the emergence of topological superconductivity requires the right balance of magnetic and spin-orbit interactions. As these interactions cannot be tuned at will, the absence of a moiré pattern decreases the chances of realizing TSC. Alternatively, the contrast between CrCl_3_ and CrBr_3_ may arise from the different magnetization directions — in-plane for CrCl_3_, out-of-plane for CrBr_3_. For the typical proposal to establish topological superconductivity, the effects of magnetic and spin-orbit interactions should be oriented perpendicularly^27,28^. In the case of planar heterojunctions, the Rashba effect from the interface acts in the plane, perpendicular to the electron momenta. Hence, for a magnetization that is directed out-of-plane, the requirement for perpendicularity is satisfied for all in-plane momenta. In contrast, an in-plane magnetization direction is only perpendicular to certain momenta. This complicates the model and is expected to have a detrimental effect on the existence of topological superconductivity. In an alternative proposal, *nodal* topological superconductivity is engineered by combining superconducting NbSe_2_ with in-plane ferromagnetism^29^. However, a problem with this approach (as realized by the authors themselves) is the negative influence of Rasbha spin-orbit interactions on the nodal state. The Rasbha effect weakens the chiral symmetry that protects the nodal states, leading to additional restrictions on the magnetic field direction. Furthermore, any protection by chiral symmetry would be further diminished by the disorder found on our CrCl_3_ islands. These arguments can explain the absence of nodal topological superconductivity in the CrCl_3_/NbSe_2_ heterostructures grown here.

In summary, we have shown the existence of a YSR state localized on the edge of the magnet/superconductor heterostructure formed by CrCl_3_ and NbSe_2_. The single monolayer islands of CrCl_3_ on NbSe_2_ are found to strongly prefer the armchair-terminated edge type and did not show a moiré pattern. Along the edge of the heterostructure, YSR states have been found, whose emergence is explained by an enhanced exchange coupling between Cr and NbSe_2_. At the origin of this enhancement is an increase in PDOS(*E*_*F*_) at the edge Cr atoms. More generally, our results demonstrate that magnet/superconductor heterostructures may exhibit edge states that are non-topological.

## Methods

### Experiments

NbSe_2_ (HQGraphene) was cleaved in vacuum (<5 × 10^−8^ mbar, fast entry lock) and subsequently degassed in UHV (<1 × 10^−9^ mbar) at 300 °C. CrCl_3_ (anhydrous, 99.99%, Sigma-Aldrich) was evaporated by e-beam evaporation (EFM3, FOCUS GmbH) onto a heated NbSe_2_ substrate (260 °C). The total evaporation time was 6 min, after which the sample was annealed for 30 min at the growth temperature. STM experiments are performed at 360 mK using an UNISOKU USM1300. Differential conductance (dI/dV) experiments were performed using standard lock-in detection with *f* = 973 Hz and *V*_*ac*_ = 60 *μ*V_*p*−*p*_ unless stated otherwise.

### Theoretical calculations

Theoretical calculations were performed using the Density Functional Theory code SIESTA, which is based on a numerical localized atomic orbital basis set and a pseudopotential description of the core electrons^23,24^. Spin-orbit coupling, within the off-site approximation, was included in all calculations^30^. To properly describe the strongly correlated electrons in the 3d orbitals of Cr, the calculations were performed within the DFT+U formalism implemented in SIESTA^31,32^. The corresponding parameters were set to *U* = 3 eV and *J* = 0.5 eV, in agreement with other works^33^. For the exchange-correlation potential the DRSLL functional was used from the vdW-DF family of functionals, which also includes nonlocal interactions to properly describe the long-range van der Waals (vdW) interactions between the monolayers^34,35^. Fully-relativistic pseudopotentials were taken from the Pseudo Dojo project in the PSML format, generated with v0.4 of ONCVPSP with the PBE GGA functional and standard accuracy^36–38^. The basis set for each species was generated automatically by SIESTA, using a DZP basis size, an energy shift of 0.01 Ry, a split norm of 0.15 and a soft confinement with a soft inner radius of 0.9 and soft confinement potential of 40 Ry, which is in agreement with the defaults of the 5.0.0-beta1 release. During relaxation, the Brillouin Zone was sampled using a regular grid of 6 × 2 × 1 k-points, with 6 points along the direction parallel to the ribbon, 2 points along the in-plane direction perpendicular to the ribbon and 1 point along the out-of-plane vacuum direction. The electronic occupations were smeared using a Fermi-Dirac distribution with a temperature of 10 meV. For the real-space grid a mesh cutoff of 800 Ry was used. For all relaxations, a maximum force threshold of 0.04 eV/Å was used for optimizing atomic positions, and a maximum stress threshold of 0.1 GPa for optimizing the unit cell vectors. To compute the density of states (DOS), the ground state electron density was first re-computed using a finer sampling of the Brillouin Zone on a 10 × 2 × 1 grid, where the directions are the same as before. Afterwards, an even finer sampling of reciprocal space on a grid of 414 × 72 × 1 was used for the actual calculation of the DOS. The states for the DOS were broadened using a Gaussian broadening with a peak width $w=\sigma \sqrt{2}=20\,{\rm{meV}}$. All atomic magnetic moments and charges were determined using a Mulliken population analysis. The AiiDA package was used to manage the simulations and keep a full record of the provenance^39,40^. The results were post-processed with the sisl package^41^.

Starting from the ground-state DFT results, the isotropic exchange *J* between pairs of atoms was calculated using the TB2J package^25^. The conventions of the Heisenberg model were chosen such that a positive *J* favors ferromagnetic alignment between two atoms. During the calculation, a reciprocal space grid of 3 × 1 × 1 was used, where the directions are the same as for the DFT calculations. For the energy integrations, a discretization was used with 80 points. Only Cr and Nb atoms were included when computing the exchange *J*’s.

To better understand the origin of the experimentally observed in-gap states, DFT calculations were performed to compute the electronic and magnetic properties of the edge of the CrCl_3_ islands. The NbSe_2_ substrate was modeled using a single monolayer. To model the edge of the CrCl_3_ islands, a nanoribbon was used with a width of 13 Cr atoms from edge to edge. The ribbons were constructed with the armchair edge, in agreement with experiment. Periodic repetitions of the ribbon were separated, in the in-plane direction perpendicular to the ribbon, by empty substrate with a width equivalent of 7 Cr atoms, which equates to about 24 Å. In the out-of-plane direction, a vacuum of around 29 Å was used. Before putting ribbon and substrate together, the atomic positions and unit cell vectors of isolated NbSe_2_ and CrCl_3_ monolayers were relaxed. The optimized lattice parameters were *a* = 3.49 Å for NbSe_2_ and *a* = 6.06 Å for CrCl_3_. To better match the lattice parameters, the CrCl_3_ was stacked with its lattice vector along a direction rotated 30^∘^ with respect to the lattice vector of NbSe_2_. Along this direction the smallest periodic length of NbSe_2_ was 6.05 Å. To make the layers match exactly, the CrCl_3_ was compressed by 0.26%. This seemed justified due to the absence of a moiré pattern in the experiments, suggesting that there is a lattice match between the two layers. Moreover, since the CrCl_3_ is grown on the NbSe_2_ substrate, it is expected that the CrCl_3_ adjusts to the substrate. Due to the anisotropy of CrCl_3_ the ribbon has two distinct edges, that are characterized by the position of the outermost Cl atoms, either up or down.

## Supplementary information

Supplementary Information

## Data Availability

The experimental data used to create the figures in this manuscript has been published (10.24416/UU01-101811) and can be downloaded from https://public.yoda.uu.nl/science/UU01/101811.html.

## Code availability

SIESTA is open-source software, available from https://siesta-project.org/siesta/. Calculation routines and results are available from Materials Cloud, via 10.24435/materialscloud:1b-aa.
