# Supplementary material for: One dimensional edge localized YSR states in CrCl3 on NbSe2
Source: NPJ Quantum Mater. 2025 May 27;10(1):51. doi: 10.1038/s41535-025-00759-2 (PMC12116383; doi:10.1038/s41535-025-00759-2)
Supplement: Supplementary file 1 — Supplementary Information [file 41535_2025_759_MOESM1_ESM.pdf]

Supplementary Information for "One Dimensional Edge Localized YSR States in $CrCl_3$ on $NbSe_2$"

Jan P. Cuperus, Arnold H. Kole, Andrés R. Botello-Méndez,
Zeila Zanolli,* Daniel Vanmaekelbergh, and Ingmar Swart†
Condensed Matter and Interfaces, Debye Institute for Nanomaterials Science, Utrecht University, The Netherlands

SUPPLEMENTARY FIGURES

Wide bias range STS experiments

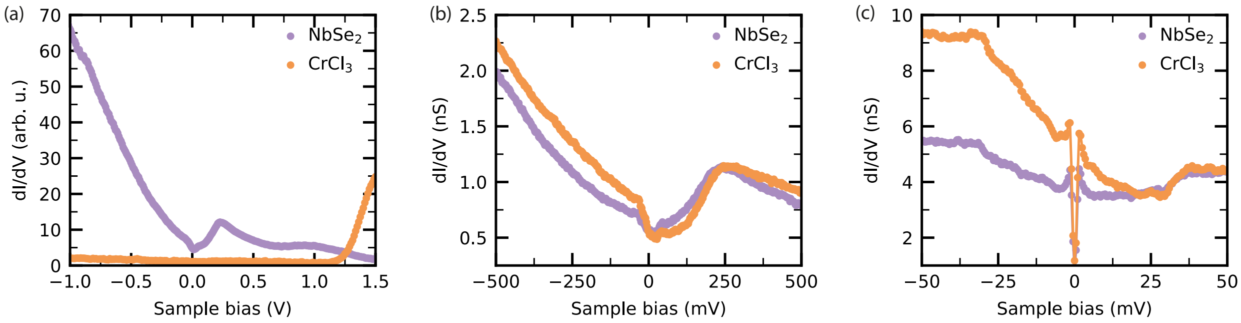

FIG. S1. **Electronic characterization of $CrCl_3/NbSe_2$ by differential conductance spectra in various bias ranges.** Spectra measured within the insulator band gap of $CrCl_3$ (b, c) probe the effect of $CrCl_3$ on the electronic properties of $NbSe_2$. (**a**) Large-range bias spectroscopy data showing the insulating nature of $CrCl_3$. The position of the $CrCl_3$ conduction band matches the observation of ref. [1] for small $CrCl_3$ flakes on $NbSe_2$. Set points: 2.0 V, 500 pA for $NbSe_2$ ($V_{ac} = 18\,\mathrm{mV_{p-p}}$), 1.5 V, 200 pA for $CrCl_3$ ($V_{ac} = 20\,\mathrm{mV_{p-p}}$). (**b**) On $CrCl_3$, the Nb d-band at ca. +250 meV is shifted to a higher energy by ca. 25 meV. Set point: 0.5 V, 300 pA ($V_{ac} = 5\,\mathrm{mV_{p-p}}$). (**c**) Spectra on $CrCl_3$ and $NbSe_2$ both show kinks at ±35 meV, suggesting that the charge-density wave of $NbSe_2$ is sustained underneath the $CrCl_3$ islands. Set point: 0.1 V, 400 pA ($V_{ac} = 1\,\mathrm{mV_{p-p}}$).

Existence of YSR state on all edges

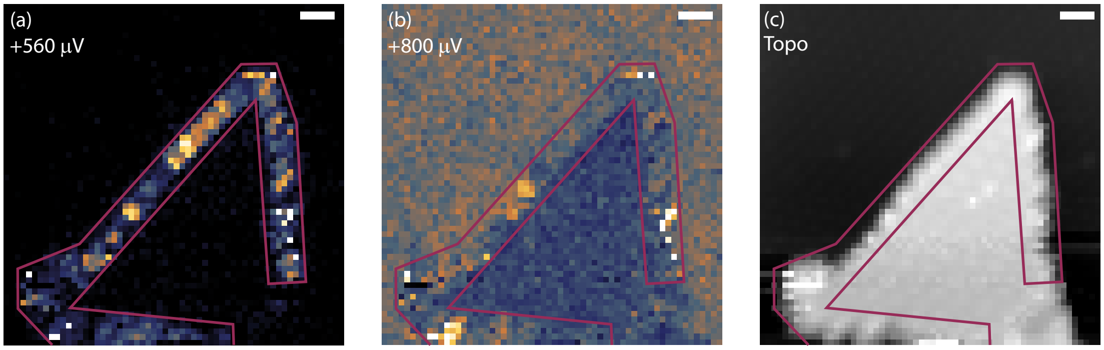

FIG. S2. **Grid spectroscopy data showing the existence of the YSR edge state on all edges.** (**a**, **b**) dI/dV signal at +560 µV and +800 µV, respectively. (**c**) Topography image acquired simultaneously with (**a**) and (**b**). The edge state is outline in (a); this outline is copied onto (**b**) and (**c**). In the bottom right corner, no YSR state signal is measured, because the $CrCl_3$ island is merged with a small $CrCl_2$ island. Scale bars: 2 nm.

Additional line spectrum on $CrCl_3$ edge

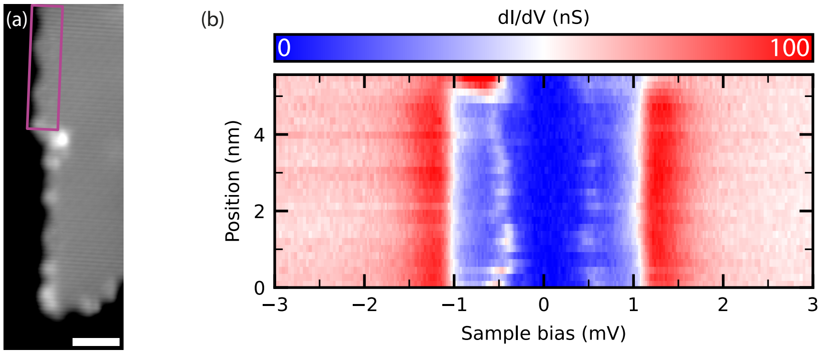

FIG. S3. **STS spectroscopy along a line segment, showing a different dispersion than the data in Fig. 2b of the main text.** (**a**) STM topograph of the island edge on which the data of (**b**) was collected. The location of the line spectrum in marked by the purple box. Set point: 0.5 V, 80 pA; scale bar: 2 nm. (**b**) Contour plot of dI/dV spectra taken along the edge segment highlighted by the purple box shown in (**a**). Set point: 5 mV, 250 pA.

Alignment grid dI/dV spectroscopy

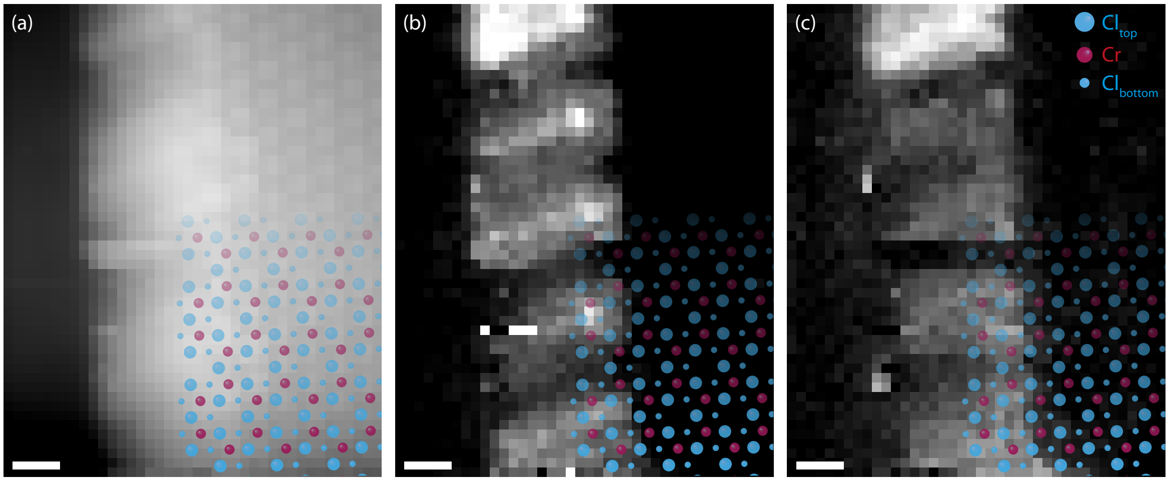

FIG. S4. **Alignment of atomic model onto YSR spatial maps using simultaneously acquired topography.** (**a**) Topography of edge segment as acquired during grid spectroscopy experiment. The periodic lattice of Cl_{top} atoms is visible and used to align the atomic model. (**b**), (**c**) Grid spectroscopy data at +560 µV (b) and −560 µV (c) showing the spatial profile of the YSR state (same data as Fig. 2c, d in main text). The atomic model from (**a**) is overlaid. Because of a double tip, the island shape in (**a**) and the spatial extent of the YSR states in (**b**, **c**) are elongated to the left. Scale bars: 5 Å.

SUPPLEMENTARY DISCUSSION

Supporting discussion of theory and simulation results

The understanding of complex systems, like the one under study in this manuscript, often requires the reduction of the full many-body correlated system into a simplified model Hamiltonian. This has indeed been the case for the study of magnetic impurities on normal metals and superconductors. In the normal state, the interaction of magnetic impurities is with conduction electrons, leading to a screening of the impurity spin. This has been successfully described by the Kondo model. In contrast, in the superconducting state the magnetic impurity breaks Cooper pairs (because of its magnetic nature), leading to localized bound states within the superconducting gap. These bound states have been successfully

explained by the Yu-Shiba-Rusinov (YSR) model. When a magnetic impurity is placed in a superconducting host, there is a competition between Kondo screening (favoring singlet formation) and the formation of YSR states (favoring a local doublet state due to pair breaking). The outcome depends on the relative magnitudes of the Kondo temperature (T_K) and the superconducting gap. In the normal state, Kondo screening dominates, and the system behaves similarly to a Kondo system. At lower temperatures, YSR states dominate, leading to bound states within the gap. Note that for the Kondo effect to occur, the conduction electron spins need to align anti-parallel to the impurity spin so that a singlet state is formed, where the total spin of the system (impurity plus conduction electrons) is minimized. Otherwise, a triplet state forms, where the total spin of the system is maximized. In this case, the impurity's magnetic moment is not screened; instead, it enhances the magnetic moment of the surrounding conduction electrons. Within the Kondo Hamiltonian, $k_B T_K \propto \exp -1/\rho|J|$ with ρ the density of states of conducting electrons at the Fermi level, and $|J|$ the magnitude of the antiferromagnetic coupling between the magnetic impurity and the conducting electrons. For $NbSe_2$ $T_K = 30K$ and $\rho \approx 2eV^{-1}$. From this, we can expect a value for $|J|$ on the order of 0.52 meV. In the superconducting state it is more difficult to estimate the density of quasiparticles, but we can expect a critical value J_c on the same order of magnitude. Note that in contrast to the Kondo effect, both FM and AFM pairing between the impurity and the quasiparticles will lead to YSR states. Using TB2J we mapped the full $CrCl_3$ nanoribbon on a $NbSe_2$ substrate system to an effective Heisenberg Hamiltonian to compute the exchange coupling J between the ribbon and the substrate. Some care has to be taken in interpreting the results. Firstly, the system is more complicated than a single transition metal magnetic impurity interacting with an elemental superconductor. Both Cr and Nb have ligands that can affect the exchange coupling. Moreover, the low expected values for J makes it difficult to get reliable values from DFT. Nevertheless, we can extract insights from the qualitative trends found from first principles.

First, we have found that the $CrCl_3$ nanoribbon has edge states close to the Fermi level, also in the presence of the $NbSe_2$ substrate, as can be seen in Fig. S5. These states have a contribution of both Cl and Cr atoms. In contrast, we find no states close to the Fermi level in the center of the ribbon. Furthermore, the computed value of the coupling J between edge impurities and conduction electrons is larger than the coupling at the center of the ribbon, and its value decays rapidly. The sign of the coupling J between Cr atoms and conduction

electrons is always positive, suggesting FM pairing, contrary to the Kondo and YSR models. However, due to the complex nature of the system and the small energies involved, it is difficult to reliably predict the sign of the pairing. Specific computational details, such as the exchange-correlation functional used, basis set, Hubbard U parameters, and the exact method used to compute the pairing, can all affect the final sign. Despite this sensitivity, we still find that the qualitative trends are robust against changes in computational details. We performed calculations with different magnetic configurations, stackings of the ribbon on the substrate, and values of the Hubbard U parameter and always found the same trends. The data to support this trend is summarized in Fig. S6. From these trends, we can say that the edge behaves differently from the center. This suggests that the conduction electrons effectively interact with a 1D chain of magnetic impurities, which could give rise to a Kondo resonance in the normal state and YSR states in the superconducting state.

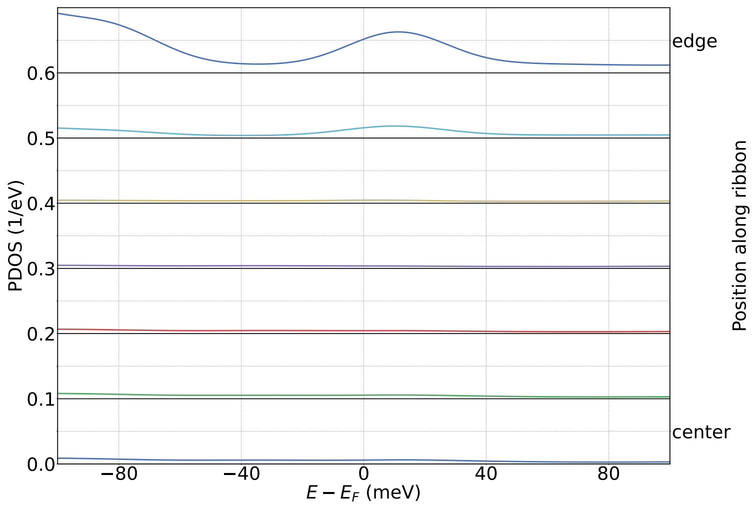

FIG. S5. **Cr projected Density of States of the nanoribbon.** Density of states, projected on the 3d orbitals of Cr (PDOS), for different Cr atoms when moving from the center of the ribbon to the edge. Note that each curve is shifted by 0.1 eV^{-1} along the y-axis with respect to the previous one in order to not overlap.

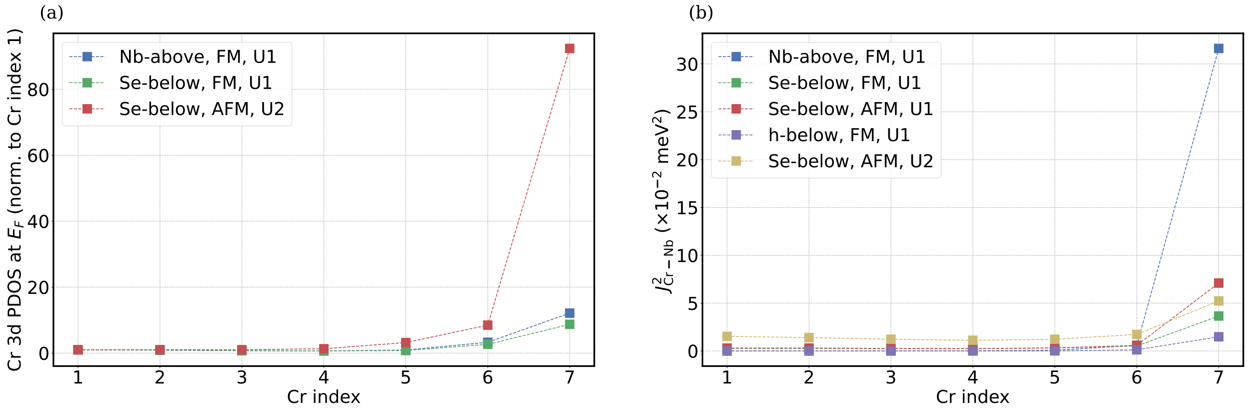

FIG. S6. **Cr 3d PDOS and exchange coupling J^2 for different geometric and magnetic Nb-Cr alignments.** Overview of (**a**) the Cr 3d density-of-states integrated over a region of width 4 meV centered on the Fermi energy and (**b**) the Cr-Nb exchange coupling J^2 for a path moving from the center to the edge of the ribbon for different stackings, magnetic alignment of Nb-Cr atoms (ferromagnetic (FM) or antiferromagnetic (AFM)) and Hubbard U parameters. For a description of the Cr index please refer to Fig. 3 from the main text. The stackings are described by a label of the form X-above or X-below where X = Nb, Se, h(ollow) refers to the site above which the Cr atom was stacked and above/below refers to whether the $CrCl_3$ was stacked above or below the $NbSe_2$, which leads to different stackings due to the inversion of the Cl ligands. For the Hubbard U parameters we tried two options, (i) U(Cr) = 3 eV, J(Cr) = 0.5 eV, U(Nb) = J(Nb) = 0 eV, which we denote as U1, and (ii) U(Cr) = 4.76 eV, J(Cr) = 0 eV, U(Nb) = 3.1 eV, J(Nb) = 0 eV which we denote as U2. Note that the qualitative trend, enhanced PDOS(E_F) and J^2 at the edge compared to the center, is always the same.

* z.zanolli@uu.nl

† i.swart@uu.nl

[1] Lu, S. *et al.* Controllable dimensionality conversion between 1D and 2D $CrCl_3$ magnetic nanostructures. *Nature Communications* **14**, 2465 (2023).
